# Building a research agenda on preventing and addressing sexual assault and intimate partner violence against trans people: a two-stage priority-setting exercise

**DOI:** 10.1186/s12961-024-01245-0

**Published:** 2024-12-18

**Authors:** Janice Du Mont, Rachel Cheung, Joseph Friedman Burley, Sarah Daisy Kosa, C. Emma Kelly, Brittany A. E. Jakubiec, Sydney Brouillard-Coyle, Sheila Macdonald

**Affiliations:** 1 Women’s College Research Institute, Women’s College Hospital, 76 Grenville St, Toronto, ON M5S 1B2 Canada; 2 Dalla Lana School of Public Health, University of Toronto, 155 College St, Toronto, ON M5T 3M7 Canada; 3 Ontario Network of Sexual Assault/Domestic Violence Treatment Centres, 76 Grenville St, Toronto, ON M5S 1B2 Canada; 4 Egale, 120 Carlton Street, Suite 217, Toronto, ON M5A 4K2 Canada; 5 Trans Wellness Ontario, 1435 Tecumseh Rd E, Windsor, ON N8W 1C2 Canada

**Keywords:** CHNRI method, Health equity, Human rights, Intimate partner violence, Sexual assault, Stakeholder engagement, Transgender

## Abstract

**Background:**

Transgender (trans) people experience high rates of sexual assault (SA) and intimate partner violence (IPV) and seldom receive the care and supports they need post-victimization. However, there is little to no research that aids in the development or improvement of related interventions. We undertook a study to build a novel Canadian research agenda on SA/IPV against trans people to guide future work and address these profound gaps in knowledge.

**Methods:**

Guided by the Child Health and Nutrition Research Initiative (CHNRI) method for research priority-setting, we developed and circulated two consecutive surveys to a multi-stakeholder group of government decision makers; mental health, health and social service providers, researchers and trans communities, among others, who proposed research questions related to preventing and addressing SA/IPV against trans persons. The initial survey launched March 2021 garnered responses from 213 stakeholders. These items were cleaned and collated into 20 final questions that fell within seven thematic areas. The refined research questions were evaluated in August 2021 on predefined criteria for answerability, feasibility, impact and equity by 79 of 95 survey 1 respondents who agreed to participate in the second survey (response rate = 83.2%). The questions were ranked using a research priority score calculated by dividing the sum of all the answers for each question across the four criteria by the number of answers received.

**Results:**

All questions were highly rated on each individual criterion and each had an overall research priority score of above 80%, with the most highly ranked question falling within the theme, “improving quality and implementation of education and training: How can training (e.g., for university/college students, educators, nurses, physicians, social workers, police, lawyers, security guards) be improved to better support trans survivors of sexual assault and intimate partner violence?”.

**Conclusions:**

These questions form Canada’s first research agenda on SA/IPV against trans people. Together, they reflect the insights of stakeholder groups who have been historically excluded from research priority-setting processes and will guide future and much-needed work on the topic. Actionable information on preventing and addressing SA/IPV against trans persons will help reduce negative outcomes associated with being victimized.

**Supplementary Information:**

The online version contains supplementary material available at 10.1186/s12961-024-01245-0.

## Background

Globally, transgender (trans) persons—individuals whose gender identity does not fully or in part align with their assigned sex at birth and includes Two-Spirit, genderqueer and non-binary identities, among others—experience elevated rates of violence and victimization [1, 2]. Among the numerous forms of violence that impact trans populations, sexual assault and intimate partner violence (SA/IPV) are particularly pervasive. According to a recent systematic review and meta-analysis [3], trans persons experience IPV at rates 1.7 times higher than cisgender individuals—those whose gender identity aligns with their assigned sex at birth. When limited to physical or sexual IPV, disparities increase to 2.2 times and 2.5 times, respectively, and persist in comparisons between trans persons and cisgender women specifically [3]. In a recent Canadian population-based survey, over one-in-four trans persons reported experiencing sexual assault in the past 5 years and three-in-five trans women reported experiencing intimate partner violence since the age of 16 [4, 5].

SA/IPV can have devastating health, social and economic impacts for trans people. These include chronic physical health problems, psychological distress, depressive disorders, suicidality, substance abuse, social isolation, housing instability and employment issues, among others [6–8]. These consequences can be compounded by concurrent experiences of stigma, discrimination and structural violence associated with cissexism, heterosexism, racism, classism, ableism and other intersecting axes of oppression [9]. When trans survivors of SA/IPV attempt to access care and support services following victimization, they frequently encounter barriers [10, 11]. Across healthcare, social service and criminal-legal sectors, these survivors are often confronted with discriminatory providers, stigma and a myriad of other institutional barriers (e.g., binary gender intake forms, exclusionary gender-specific service mandates) [10, 12].

Research that aids in the development or improvement of interventions to prevent and address SA/IPV against trans people is therefore essential. However, much of the research, advocacy and interventions on SA/IPV has been conceptualized in cis- and hetero-normative binaries that reduce the dynamics of gender-based violence to unilateral abuse perpetrated by cisgender heterosexual men against cisgender heterosexual women [13]. The assumption that gender-based violence exclusively takes this form perpetuates the erasure of SA/IPV against trans people and limits the collection of trans-specific data in prevailing research on the subject [13, 14]. Insofar as trans survivors of SA/IPV have been included in research, it has largely been in the context of studies with small convenience samples that pool data on violence against lesbian, gay, bisexual, transgender and queer persons [10, 14]. This homogenization can obscure important within-group differences in experiences of SA/IPV and, in doing so, further contribute to the erasure of trans survivors [10, 13, 14]. These gaps in research are especially evident in Canada, where to date only two population-based studies have ever included any focus on SA/IPV against trans people [4, 5, 15].

With the recent establishment of the 2SLGBTQI + secretariat and national 2SLGBTQI + Action Plan, the Canadian government has committed to supporting research and programs that “improve social, economic, and health outcomes for 2SLGBTQI + Canadians” and specifically identified “strengthen[ing] 2SLGBTQI + data and evidence-based policy making” as a priority [16]. This support should include rigorous and action-oriented research into SA/IPV against trans Canadians; however, no research agenda currently exists to guide funding mandates and ensure that they reflect the perspectives of relevant Canadian researchers, service providers, policymakers and trans individuals themselves.

In response to the dearth of existing Canadian literature on this topic and call to action by the federal government, we undertook a study to build Canada’s first research agenda on preventing and addressing SA/IPV against trans people.

## Methods

This study employed a systematic, validated and collaborative method developed by the Child Health and Nutrition Research Initiative (CHRNI) [17]. Publicly available and increasingly used in research priority-setting processes globally, this approach allows researchers, policymakers, funders and other relevant stakeholders to participate in identifying and evaluating diverse research options in a given area [18–22]. The CHNRI method is inherently flexible in both the criteria utilized for priority-setting and the context to which it may be applied. As such, the method was adapted in this study to “meet the specific needs of [our] priority–setting exercise,” as recommended by its developers [21, p1]. The method includes recommended steps for the determination of research priorities, of which the following guided the present study: establishing a management group, defining the context and scope of the research, listing and selection of evaluation criteria and questions, generating research questions, collating and refining questions and evaluating and ranking research questions.

Our management group, established to design and oversee the execution of the research priority-setting project, included an international expert in gender-based violence among diverse populations (J.D.M.), a global leader in responding to SA/IPV in healthcare settings (S.M.), a health research methodologist with extensive experience in violence research (S.D.K.) and a researcher focused on queer and trans health and human rights policy (B.J.). This group has a history of collaborating to enhance trans-affirming post-assault care among health and social service providers and creating and implementing related surveys [23–26].

The research of interest was defined to include the full continuum of information that could aid in improving responses to SA/IPV against trans persons. Research questions generated within this broad scope could encompass, for example, the frequency and determinants of SA/IPV in trans communities, how SA/IPV may impact specific trans populations (e.g., racialized), as well as the design or evaluation of interventions. Relevant criteria for the evaluation of research questions were determined through synthesis and modification of the criteria applied in 12 previous studies on or using the CHNRI method (e.g., answerability, effectiveness, deliverability, impact, equity) [17–20, 22, 27–33]. The final list of criteria and corresponding questions can be found in Table 1.

**Table 1:** Evaluation criteria and questions

Criteria	Questions
Answerability	Is the question clear and could research be designed to answer it? [Adapted from 17, 22, 27, 28, 31]
	Would research arising from the question be ethical? [Adapted from^17^, ^19^, ^20^, ^22^, ^29^–^31^, ^33^]
Feasibility	Could research arising from the question be completed within a reasonable time frame of 3–5 years? [Adapted from 18, 28, 31, 32]
	Would research associated with this question be fundable? [Adapted from 17, 31]
	Would the cost required for this research be reasonable? [Adapted from 17, 19, 22, 29, 31]
Impact	Would the results of the research fill an important knowledge gap? [Adapted from 28]
	Could the results of the research lead to an effective intervention or program? [Adapted from^17^, ^19^, ^20^, ^22^, ^29^]
	Could the results of the research lead to improvements in policy? [Adapted from 18, 22]
Equity	Would the results of the research benefit diverse members of trans communities? [Adapted from 17, 18, 20, 22, 28–30]

### Defining research priority-setting stakeholders

The characteristics of research priority-setting experts were defined to ensure the inclusion of a diverse array of stakeholder groups from across Canada with relevant professional and/or lived experience/expertise in SA/IPV against trans people [19, 22]. To capture the experiences of all individuals for whom the research might be significant, priority-setting stakeholders included academics/researchers, government decision-makers/policymakers, advocates, health and social service providers, representatives of professional associations, funders, trans community members and survivors.

### Measures

Two consecutive surveys were developed by the management group for stakeholder engagement; the first to identify priority research questions (survey 1) and the second to evaluate them (survey 2).

Both survey 1 and survey 2 were piloted by a research and evaluation working group composed of trans community members and allies with relevant research, policy and/or practical expertise in violence prevention and care/support (see Acknowledgements for working group membership). Members of the working group completed the surveys and then convened to provide feedback on clarity, tone, flow and formatting. Suggested revisions, which centered on increasing the visual accessibility of the surveys and improving the clarity of instructions, were addressed and a final version of the two surveys was produced.

Both surveys were prefaced by a letter of information and consent form, which outlined the purpose of the surveys, eligibility criteria, participant responsibilities, potential harms and benefits of participation and important information on confidentiality. To complete the surveys, participants had to live in Canada, be aged 18 years or older, be able to read/write comfortably in English, be able to provide informed consent and belong to one of our predefined stakeholder groups.

#### Survey 1: generating research questions

Survey 1 included three sections. Section 1 collected sociodemographic information, including age (18–24, 25–34, 35–44, 45–59, 60 + , prefer not to answer), gender (woman, man, cisgender, transgender, bigender, transfeminine, transmasculine, genderqueer, agender, non-binary, gender fluid, Two-Spirit, a gender not listed above [please specify], prefer not to answer), sexual orientation (lesbian, gay, bisexual, queer, Two-Spirit, pansexual, asexual, heterosexual, an orientation not listed above [please specify], prefer not to answer), ethnicity/racial background most identified with [34] (Arab, West Asian [e.g., Iranian, Afghan], Black, Chinese, Filipino, Indigenous [e.g., Inuk, Métis, First Nations], Japanese, Korean, Latin American, Southeast Asian [e.g., Vietnamese, Cambodian, Laotian, Thai], South Asian [e.g., East Indian, Pakistani, Sri Lankan], white, other, a background not listed above [please specify], prefer not to answer), highest level of education completed (professional degree [e.g., MD, LLB, MBA], doctorate, master’s, bachelor’s, college, high school diploma or high school equivalent, less than a high school diploma or equivalent, prefer not to answer), province/territory of residence (British Columbia, Alberta, Saskatchewan, Manitoba, Ontario, Quebec, New Brunswick, Nova Scotia, Prince Edward Island, Newfoundland and Labrador, Nunavut, Northwest Territories, Yukon) and geographical area (urban, rural, suburban, remote).

Section 2 collected information on membership in at least one research priority-setting stakeholder group (research/academia, government/policy, advocacy, healthcare, social services, professional associations, foundations/funding agencies, transgender communities, sexual assault/intimate partner violence survivors, another stakeholder group not listed above [please specify], prefer not to answer) and the type of experience respondents felt primarily informs their expertise in the area (professional experience, lived experience, both, prefer not to answer). Finally, Section 3 asked respondents to draw on their lived and/or professional experience to generate three to five priority research questions that they believed could aid in better addressing and preventing SA/IPV against trans people. A final question asked respondents about their willingness to participate in a subsequent survey to rate research questions generated in response to survey 1 findings. Those who responded “yes” were redirected to a separate page where they could input their email address for future contact. These email addresses were not linked to survey 1 responses.

#### Survey 2: evaluating research questions

Survey 2 contained three sections. Sections 1 and 2 collected the same sociodemographic data and information on expertise as that collected in survey 1. Section 3 presented respondents with a finalized list of 20 priority research questions refined from survey 1 and asked them to rate each according to predetermined criteria (see Table 1). Respondents answered each of the evaluation questions as follows: yes (meets criteria), no (does not meet criteria), maybe (not sure meets criteria) and don’t know (insufficient knowledge to assess criteria).

### Procedure

To reach a diverse stakeholder group, we circulated open invitations to complete survey 1 through multiple channels. Dissemination efforts were undertaken through: (1) social media (Twitter and Facebook posts); (2) relevant organizational listservs, newsletters and websites (e.g., the Sexual Violence Research Initiative, Egale, the Canadian Forensic Nurses Association); and (3) community networks and other channels focused on SA, IPV and/or trans issues (e.g., trans-LINK Network member organizations) [35]. All recruitment materials contained brief information about the study, including eligibility criteria and an invitation to complete the 5 to 7 min survey on the Qualtrics platform. The survey was launched 25 March 2021 and remained open for approximately 12 weeks, with biweekly reminders disseminated via email, Twitter and Facebook.

Email invitations for survey 2 were sent to those stakeholders who agreed to evaluate questions on completion of survey 1. The survey was launched on 23 August 2021 and remained open for approximately four weeks, with weekly email reminders.

### Analysis

In total, survey 1 respondents submitted 464 unique items, 25 of which required separation into two or more different items (*n* = 36). From the 500 resulting items, those that were not questions (*n* = 23), contained no mentions of SA/IPV against trans persons (*n* = 206), were not otherwise relevant (e.g., “Why should the rate of sexual assault against trans people be studied separately from the rate of sexual assault against cis people?”; *n* = 38) were first removed, then items deemed too narrow (e.g., “At what age did you first experience sexual assault?”; *n* = 87) or too broad (e.g., “Which voices have been historically erased in conversations about sexual assault and intimate partner violence?”; *n* = 31), leaving 115 research questions. These questions were then coded based on content, with discrepancies in coding resolved on a case-by-case basis through mediated group discussion (J.D.M., R.C., J.F.B). This resulted in the development of 13 preliminary categories of questions: access to supports, aftermath of assault, alternative models of response, defining experiences of assault, direct care and supports, education and training, information and prevention, policies and protocols, prevalence, reporting and disclosure, resources, risk and protective factors and root causes. Questions situated within each of the 13 categories were examined for and placed in order by similarity in content and, where questions overlapped, a single new question was drafted by members of the research team to capture their content (J.D.M., J.F.B., R.C.). For example, questions “How can we better reach into trans, non-binary and Two-Spirit community to share knowledge on IPV so it reaches those who need more information?”, “Where do trans folks first learn about what sexual assault and intimate partner violence are? Are there additional settings where such education could be provided earlier in life?” and “How can we better design support resources and tools to reach neuroatypical trans and non-binary population when it comes to sexual assault and intimate partner violence?” were collated into a single question, “How can existing information and resources about sexual assault and intimate partner violence be improved and made more accessible across a variety of settings for trans people (e.g., different cognitive abilities)?” (J.D.M., J.F.B., R.C.). Twenty questions remained after the initial 115 were collapsed and redrafted following this process. From the 20 final questions, 7 themes were developed, reflecting a consolidation of the 13 preliminary categories. The final 20 priority questions for inclusion in survey 2 were then reviewed and approved by all members of the management group, with very subtle changes to wording incorporated as appropriate.

Each of the four criteria used to evaluate candidate research questions presented in survey 2 were assigned scores calculated by dividing the sum of all the informed (i.e., non-blank) answers for that criterion (“yes = 1,” “no = 0,” or “unsure = 0.5”) by the total number of informed answers received. Overall research priority scores were calculated by dividing the sum of all the answers for each question across the four criteria by the number of answers received. Average expert agreement was calculated by first dividing the number of the experts who provided the most frequent answer to each criterion by the number of experts who scored that criterion, then summing the result for all four criteria and dividing by the number of criteria (four). Research questions were ranked by research priority score. For calculation of criterion scores, research priority scores and average expert agreement, blanks were left out of the calculation in both numerator and denominator, rounding to one decimal place only occurred after all computations (including ranking) were complete and the scores are presented as a percentage.

## Results

There were 213 respondents to survey 1. Of these, 95 indicated that they would participate in survey 2, 79 of whom completed the second survey (response rate = 83.2%).

### Sociodemographics

The most frequently reported age range in the respondent sample was 25 to 34 in survey 1 (35.2%) and survey 2 (35.1%). In surveys 1 and 2, respondents commonly identified as women (64.3% and 68.8%, respectively), non-binary (21.6% and 11.7%), transgender (13.6% and 18.2%), transmasculine (10.8% and 10.4%) and/or genderqueer (10.3% and 9.1%). Approximately two fifths of respondents identified as heterosexual (38.0% and 40.3%), one third as queer (31.9% and 31.2%) and/or one sixth as bisexual (15.5% and 16.9%). With respect to racial background/ethnicity, many respondents identified as white in surveys 1 and 2 (78.3% and 77.9%). In both surveys 1 and 2, approximately one-third of respondents had either a bachelor’s degree (34.7% and 33.8%) or a master’s degree (30.0% and 33.8%). The majority of respondents lived in Ontario, Canada (77.0% and 72.7%) and in urban settings (65.3% and 70.1%) (see Table 2).

**Table 2:** Sociodemographic characteristics of survey 1 and survey 2 respondents

Characteristics	Stakeholders who generated research questions (*N* = 213)		Stakeholders who evaluated research questions (*N* = 79)	
	*N*	%	*N*	%
Age	213		77	
18–24	18	8.5	2	2.6
25–34	75	35.2	27	35.1
35–44	45	21.1	13	16.9
45–59	58	27.2	22	28.6
60 +	14	6.6	12	15.6
Prefer not to answer	3	1.4	1	1.3
Gender*	213		77	
Woman	137	64.3	53	68.8
Man	20	9.4	9	11.7
Cisgender	39	18.3	13	16.9
Transgender	29	13.6	14	18.2
Bigender	2	0.9	1	1.3
Transfeminine	9	4.2	4	5.2
Transmasculine	22	10.3	8	10.4
Genderqueer	22	10.3	7	9.1
Agender	2	0.9	2	2.6
Non-binary	46	21.6	9	11.7
Gender fluid	5	2.3	3	3.9
Two-Spirit	1	0.5	0	0.0
Neutrois (Neutral gender)	1	0.5	0	0.0
Gender non-conforming woman	0	0	2	2.6
Prefer not to answer	2	0.9	1	1.3
Sexual orientation*	213		77	
Lesbian	25	11.7	7	9.1
Gay	20	9.4	4	5.2
Bisexual	33	15.5	13	16.9
Queer	68	31.9	24	31.2
Two-Spirit	1	0.5	0	0.0
Pansexual	29	13.6	13	16.9
Asexual	4	1.9	0	0.0
Demisexual panromantic	1	0.5	0	0.0
Heteroflexible	1	0.5	0	0.0
No label	1	0.5	0	0.0
Omnisexual	2	0.9	0	0.0
Heterosexual	81	38.0	31	40.3
Prefer not to answer	5	2.3	1	1.3
Primary ethnicity/racial background	212		77	
Arab	0	0.0	0	0.0
West Asian	2	0.9	0	0.0
Black	8	3.8	4	5.2
Chinese	5	2.4	1	1.3
Filipino	2	0.9	1	1.3
Indigenous	7	3.3	1	1.3
Japanese	0	0.0	0	0.0
Korean	1	0.5	0	0.0
Latin American	4	1.9	0	0.0
Southeast Asian	0	0.0	0	0.0
South Asian	11	5.2	1	1.3
White	166	78.3	60	77.9
Other (e.g., mixed race/ethnicity, Sephardic Jew, Western European)	5	2.4	7	9.1
Prefer not to answer	1	0.5	2	2.6
Highest level of education	213		77	
Professional degree	17	8.0	11	14.3
Doctorate	8	3.8	6	7.8
Master's	64	30.0	26	33.8
Bachelor's	74	34.7	26	33.8
College	28	13.1	7	9.1
High School diploma or a high school equivalent	16	7.5	0	0.0
Less than a high school diploma or equivalent	2	0.9	0	0.0
Prefer not to answer	4	1.9	1	1.3
Province/territory	213		77	
British Columbia	16	7.5	7	9.1
Alberta	2	0.9	1	1.3
Saskatchewan	4	1.9	2	2.6
Manitoba	4	1.9	3	3.9
Ontario	164	77.0	56	72.7
Quebec	12	5.6	4	5.2
New Brunswick	2	0.9	1	1.3
Nova Scotia	1	0.5	0	0.0
Prince Edward Island	1	0.5	2	2.6
Newfoundland and Labrador	2	0.9	0	0.0
Nunavut	0	0.0	0	0.0
Northwest Territories	2	0.9	1	1.3
Yukon	3	1.4	0	0.0
Geographic area	213		77	
Urban	139	65.3	54	70.1
Rural	30	14.1	14	18.2
Suburban	41	19.2	8	10.4
Remote	3	1.4	1	1.3
Stakeholder group*	207		79	
Research/academia	42	20.3	24	30.4
Government/policy	15	7.2	3	3.8
Advocacy	63	30.4	19	24.1
Healthcare	92	44.4	28	35.4
Social services	81	39.1	23	29.1
Professional associations	35	16.9	13	16.5
Foundations/funding agencies	5	2.4	1	1.3
Transgender communities	66	31.9	24	30.4
Sexual assault/intimate partner violence survivors	76	36.7	26	32.9
Queer communities	1	0.5	0	0.0
Other: e.g., military, peer-support/mentorship	1	0.5	2	2.5
Prefer not to answer	0	0.0	0	0.0
Experience informing expertise	207		79	
Professional experience	86	41.5	37	46.8
Lived experience	30	14.5	14	17.7
Both	89	43.0	28	35.4
Prefer not to answer	2	1.0	0	0.0

^*^Categories are not mutually exclusive

Various stakeholder groups were represented in both surveys 1 and 2: the largest groups were healthcare (44.4% and 35.4%), social services (39.1% and 29.1%), sexual assault/intimate partner violence survivors (36.7% and 32.9%), transgender communities (31.9% and 30.4%), advocacy (30.4% and 24.1%) and/or research/academia (20.3% and 30.4%). Respondents reported several types of experience informing their expertise in both surveys: professional (41.5% and 46.8%), lived (14.5% and 17.7%) and both (43.0% and 35.4%) (see Table 2).

### Research priorities

Research priority scores and average expert agreement, respectively, were high for all items (≥ 83%, ≥ 75%).

Within theme 1, *Defining the scope of the problem*, there were four questions, one of which was ranked among the top 10 research priorities: “What are the effects (i.e., impacts, recovery) of experiencing SA/IPV for trans survivors of varying social locations (e.g., transmasculine, transfeminine, disabilities, living situation)?” (rank of 8).

Within theme 2, *Increasing understanding of contextual and contributing factors,* there were three questions, one of which was ranked in the top 10: “What factors are related to diverse trans survivors (e.g., Black, Indigenous and people of colour [BIPOC], transfeminine, transmasculine) leaving a situation of SA/IPV (e.g., housing, finances, social supports)?” (rank of 10).

Both questions within theme 3, *Expanding knowledge of disclosure and reporting* ranked in the top 10: “What factors impact diverse trans survivors’ disclosure of SA/IPV in healthcare and other support settings (e.g., BIPOC, living in a rural area)?” (rank of 2) and “How has the criminal justice system (e.g., police) responded to reports of SA/IPV by trans survivors?” (rank of 6).

Within theme 4, *Enhancing accessibility and appropriateness of supports*, there were seven questions, three of which ranked in the top 10: “What qualities/features of providers and services are important to diverse trans people (e.g., BIPOC, sex workers, low income) seeking support after SA/IPV?” (rank of 3), “How can 2SLGBTQIA + and SA/IPV services be more responsive to the culture and needs of diverse trans communities and survivors (e.g., BIPOC, queer, sex work positive)?” (rank of 5) and “What barriers and facilitators impact access to hospital, health, social (shelters) and/or legal services for trans survivors of SA/IPV (e.g., social identity, psychosocial circumstances, systems of oppression)?” (rank of 9).

Within theme 5, *Improving quality and implementation of education and training,* there were two questions, both of which ranked in the top 10: “How can training (e.g., for university/college students, educators, nurses, physicians, social workers, police, lawyers, security guards) be improved to better support trans survivors of SA/IPV?” (rank of 1) and “How can training that facilitates cultural competence and the provision of appropriate care (e.g., attention to gender and race) to trans survivors of SA/IPV be embedded in academic and workplace settings?” (rank of 7).

A single question comprised theme 6, *Developing alternative models of response* and was ranked 4th: “What community-based models of emergency and crisis care can be developed to better respond to trans survivors of SA/IPV (i.e., as opposed to law enforcement models)?”.

A single question also comprised the 7th and final theme, *Advancing multi-level interventions for prevention*: “What interpersonal and community interventions can be improved or newly developed to better prevent SA/IPV against diverse trans people (e.g., BIPOC, living in rural areas, disabilities)?” and ranked 12th as a research priority (see Table 3 for listing of questions under each theme).

**Table 3:** Research priorities for preventing and addressing sexual assault and intimate partner violence against transgender people

Theme and questions	Rank	RPS^1^%	A^2^%	F^3^%	I^4^%	E^5^%	AEA^6^%
Defining the scope of the problem							
How do trans people define what constitutes intimate partner violence?	13	86.4	91.8	87.0	81.6	85.2	79.7
How common are sexual assault and intimate partner violence among groups with different gender identities (e.g., cisgender women, transmasculine, transfeminine)?	11	86.8	91.7	84.2	85.3	85.9	78.8
What are the characteristics of incidents (e.g., survivors, perpetrators, settings) of sexual assault and intimate partner violence against trans people?	18	83.4	88.0	82.6	82.7	80.3	74.9
What are the effects (i.e., impacts, recovery) of experiencing sexual assault and intimate partner violence for trans survivors of varying social locations (e.g., transmasculine, transfeminine, disabilities, living situation)?	8	87.7	93.0	82.4	87.1	88.2	81.2
Increasing understanding of contextual and contributing factors							
What are the root causes of sexual assault and intimate partner violence against trans people (e.g., stigma, negative stereotypes and other implicit biases)?	19	83.2	89.9	80.2	81.0	81.6	74.8
What factors are associated with experiencing sexual assault and intimate partner violence as a trans person (e.g., sociodemographic characteristics, disclosure of gender identity, undergoing gender-affirming surgery/medical transition, use of dating apps)?	15	85.1	89.2	82.6	82.2	86.3	78.3
What factors are related to diverse trans survivors (e.g., BIPOC, transfeminine, transmasculine) leaving a situation of sexual assault or intimate partner violence (e.g., housing, finances, social supports)?	10	87.0	93.6	78.9	86.2	89.5	80.0
Expanding knowledge of disclosure and reporting							
What factors impact diverse trans survivors’ disclosure of sexual assault and intimate partner violence in healthcare and other support settings (e.g., BIPOC, living in a rural area)?	2	91.3	98.1	87.4	88.8	91.0	87.4
How has the criminal justice system (e.g., police) responded to reports of sexual assault and intimate partner violence by trans survivors?	6	88.3	94.9	83.8	87.5	87.2	82.7
Enhancing accessibility and appropriateness of supports							
What information does the public need to better support trans survivors of sexual assault and intimate partner violence?	16	84.9	91.0	79.3	84.5	84.6	77.1
How can existing information and resources about sexual assault and intimate partner violence be improved and made more accessible across a variety of settings for diverse trans people (e.g., different cognitive abilities)?	17	83.9	88.0	77.3	84.5	85.9	74.9
What barriers and facilitators impact access to hospital, health, social (shelters) and/or legal services for trans survivors of sexual assault and intimate partner violence (e.g., social identity, psychosocial circumstances, systems of oppression)?	9	87.4	91.6	83.0	86.8	88.2	82.3
What qualities/features of providers and services are important to diverse trans people (e.g., BIPOC, sex workers, low income) seeking support after sexual assault and intimate partner violence?	3	90.4	90.8	88.7	90.9	91.0	86.9
Do trans survivors’ perceptions and experiences (e.g., barriers, misunderstood, dismissed) of post-sexual assault and intimate partner violence services vary across their differing social locations (e.g., BIPOC)?	14	86.2	89.5	83.5	86.3	85.5	82.3
How can organizations across different sectors form partnerships to effectively address the mental health needs of trans survivors seeking services for sexual assault and intimate partner violence?	20	83.2	89.7	80.4	81.7	80.8	75.0
How can 2SLGBTQIA + and sexual assault and intimate partner violence services be more responsive to the culture and needs of diverse trans communities and survivors (e.g., BIPOC, queer, sex work positive)?	5	88.7	90.0	87.4	88.1	89.5	83.2
Improving quality and implementation of education and training							
How can training (e.g., for university/college students, educators, nurses, physicians, social workers, police, lawyers, security guards) be improved to better support trans survivors of sexual assault and intimate partner violence?	1	91.4	97.4	88.1	90.9	89.2	87.1
How can training that facilitates cultural competence and the provision of appropriate care (e.g., attention to gender and race) to trans survivors of sexual assault and intimate partner violence be embedded in academic and workplace settings?	7	88.2	91.7	86.7	87.0	87.5	82.6
Developing alternative models of response							
What community-based models of emergency and crisis care can be developed to better respond to trans survivors of sexual assault and intimate partner violence (i.e., as opposed to law enforcement models)?	4	89.8	94.5	85.1	90.9	88.9	84.0
Advancing multi-level interventions for prevention							
What interpersonal and community interventions can be improved or newly developed to better prevent sexual assault and intimate partner violence against diverse trans people (e.g., BIPOC, living in rural areas, disabilities)?	12	86.4	88.9	81.0	87.0	88.9	77.3

^1^RPS represents research priority score

^2^A represents answerability

^3^F represents feasibility

^4^I represents impact

^5^E represents equity

^6^AEA represents average expert agreement

## Discussion

This research priority-setting study—an initial step toward systematically and comprehensively determining how best to address a profound dearth of knowledge on SA/IPV against trans people—is a globally unprecedented effort to attend to these public health and human rights equity issues in Canada and worldwide [36]. To effectively narrow potentially limitless research possibilities while accounting for trans perspectives, we utilized the CHNRI method, which integrates diverse stakeholders into the priority-setting process and has been proven to be a systematic, transparent, inclusive and fair approach across multiple disciplines and diverse subject areas [17, 19, 22, 31]. This method has the potential to increase research capacity while tempering the effect of any one group or person’s biases on the final research agenda [22]. Further, stakeholder engagement promotes continuity between priority-setting and implementation of research and encourages participants to take ownership of and promote research agendas within and across their networks [19]. In these regards, CHNRI was the ideal method to rigorously develop a set of research questions on SA/IPV against trans people.

While approximately 50 CHNRI priority-setting exercises had been conducted globally as of 2016 [37], the developers of the CHNRI method indicated that it “owes its uptake and implementation in a large part to its flexibility, as it can be readily tailored to many different contexts and purposes.” [21, p5-6] With the guidance of our management group, we adapted several steps of the priority-setting process to suit our context, resulting in actionable findings particular to preventing and addressing sexual assault and intimate partner violence against trans people, a novel area of focus in CHNRI methodology and a critically understudied field.

Our robust sample size of experts exceeded the 45 to 55 thought necessary by CHNRI developers to stabilize an expert group’s collective opinion on categorically expressed research questions [38] and the composition thereof reflects a unique adaptation to the inherently flexible CHNRI approach. While CHNRI studies have typically engaged clinicians, academics, professional associations and government officials as experts, our study also solicited the input of trans community members, potential end-users of the research [17, 18, 33]. Indeed, as a comparable proportion of representatives from various stakeholder groups participated in both surveys 1 and 2, diverse perspectives were integrated throughout the entire priority-setting process. Yoshida et al. [33] emphasized the importance of representation of the broader community in leveraging specialized knowledge that would not otherwise be reflected and balancing the varying emphasis that different groups place on criteria used to evaluate potential research questions. Further, by incorporating this range of perspectives, we promoted “deep inclusion” to equitably facilitate the rapid generation of practical knowledge needed to respond to the unrelenting crisis facing trans communities [39, p216]. Trans persons’ perspectives have been in particular historically absent from decision-making processes related to research activities in their own communities, a practice that has often alienated them from academia, healthcare and beyond and perpetuated their continued erasure, marginalization and exploitation [39, 40]. This novel research agenda instead brought forth trans voices to the benefit of all parties, in alignment with the principles of health equity and just priority-setting and calls for “research on transgender people [to] benefit transgender people” [40, p628].

Despite the array of groups to which stakeholders belonged, priority and average expert agreement scores for all 20 evaluated items were high, suggesting a shared emphasis on the importance of a range of research activities to better understand, address and prevent SA/IPV against trans people. The highest ranked priority (priority score: 91.4%), addressing improvements to training for various groups (see Table 3), is perhaps unsurprising given the dearth in knowledge on responses to trans survivors, as previously identified [41, 42]. However, all other questions were assigned a priority score of 83.2% or higher, which may reflect the significance of the research gaps that necessitated this study and the critical need—across disciplines, sectors and communities—for research addressing the diverse thematic areas captured by items included in survey 2. Tomlinson et al. [22, p137] have suggested that this phenomenon may also be a product of the type of research priorities evaluated; studies containing “practical questions, pertaining to prevention and intervention” could reflect optimism or urgency among the scorers as it relates to the production of actionable information.

Indeed, survey 2 questions related to prevention, intervention and improving or adapting supports ranked highly relative to other types of items in our study, which may reflect the interests of our experts, many of whom could be directly and positively impacted by such tangible research. Four of the top five questions directly related to the work of healthcare or social service providers (35.4% and 29.1% of the survey 2 sample, respectively), while none of the questions nested under “Increasing understanding of contextual and contributing factors,” for example, ranked higher than 10th place. However, it is noteworthy that many of the lower-ranked questions could provide the contextualizing information necessary to design and implement such supports and their high scores underline their continued importance. Moreover, no item scored less than 88.0% on the “answerability” criterion, so these questions’ priority scores were lowered by the other criteria, perhaps reflecting respondents’ perception that they would not be immediately impactful [22].

### Limitations

Despite the care taken to involve diverse participants in the priority-setting process, it was not possible to ensure that every voice among stakeholder groups was represented. Indeed, the sample for survey 1, which then had implications for survey 2, overrepresented individuals from Ontario and those who identified as white. Additionally, the survey may not have reached those without access to technology—such as the most marginalized members of the trans communities—and was accessible only in English. As survey 1 was distributed widely online, we were not able to calculate a response rate. There may also have been differences between the survey 1 and 2 samples in terms of their characteristics, which could indicate a potential for response bias. For example, survey 2 respondents tended to be older (60 + : 6.6% versus 15.6%), be researchers/academics (20.3% versus 30.4%) and have professional degrees (8.0% versus 14.3%). As the research questions initially generated following the CHNRI method were reflective of the sample from which they were solicited, they may not have comprehensively reflected the scope of promising avenues for further research, thereby impacting later stages of the priority-setting exercise [37]. While sampling biases are a limitation inherent to the CHNRI method [37], the profound structural marginalization of trans persons, particularly those with intersecting identities [9, 39] and the dispersion of gender affirming providers [10, 12] may have posed a particular challenge to recruitment. Further, the practical requirements of reducing a substantial variety of possible research questions to a manageable, carefully crafted selection may have introduced bias from the research team. However, the effect was minimized by carefully following a well-established method involving multiple researchers assessing responses and reaching consensus while working from existing submissions rather than generating options, thereby limiting the team’s influence.

## Conclusions

This study represents a singular contribution to research related to SA/IPV against trans people in Canada, resulting in a first-of-its-kind, comprehensive agenda to guide future research into an understudied yet vitally important subject area that reflects the insights of the individuals most impacted but infrequently included in research and priority-setting exercises. The success of future research hinges on the inclusion of diverse perspectives and insights offered by, for example, social service and healthcare workers, researchers, policymakers and, in particular, survivors of SA/IPV and trans people. As Lo and Horton [36] have asserted in their call for research on trans health, fully addressing the challenges that trans people face requires an intersectoral response. By undertaking this work, we are responding to the calls to action from trans survivors, service providers, researchers and policymakers [16, 36, 39]. This novel Canadian research agenda can help inform the efficient allocation of resources to initiatives and efforts that better prevent and address SA/IPV against trans people across Canada and offer a framework for using CHNRI methodology to engage with marginalized communities to advance research in critically understudied areas. Next steps include sharing our results with broader stakeholder groups and strategizing with them to undertake the prioritized research.

## Supplementary Information

Supplementary Material 1. Data supporting the study’s conclusions (.xslx).

## Data Availability

The data supporting the study’s conclusions are included in the manuscript and Supplementary Material 1.

## Abbreviations

2SLGBTQIA +: Two-Spirit, lesbian, gay, bisexual, transgender, queer and/or questioning, intersex, asexual and more
CHNRI: Child Health and Nutrition Research Initiative
IPV: Intimate partner violence
SA: Sexual assault
